# Bearing characteristics and parameter optimization of high-strength reticulated shell composite support for deep soft rock roadways

**DOI:** 10.1038/s41598-026-46034-7

**Published:** 2026-05-26

**Authors:** Gejun Tong, Yong Luo, Jiaping Xiao, Jianyong Pang, Heng Wang

**Affiliations:** 1 Huainan Vocational Technical College, Huainan, 232000 Anhui Province China; 2 The First Surveying Team of Anhui Charcoal Field and Geology Bureau, Huainan, 232000 Anhui Province China; 3 State Key Laboratory for Safe Mining of Deep Coal and Environment Protection, Huainan Mining (Group) Co., Ltd., Huainan, 232000 Anhui Province China; 4 School of Civil Engineering and Architecture, Anhui University of Science and Technology, Huainan, 232001 Anhui Province China; 5 Fuyang Normal University, Fuyang, 236000 Anhui Province China

**Keywords:** Deep soft rock roadway, High-strength reticulated shell composite support, Numerical simulation (ABAQUS), Similar model test, Bearing characteristic, Engineering, Materials science

## Abstract

With the increase of coal mining depth, deep soft rock roadways face the “five-high” complex environment (high erosion, high ground temperature, high osmotic pressure, high ground pressure, strong disturbance), leading to prominent problems such as severe surrounding rock deformation, floor heave, and large sidewall deformation. Traditional support structures are difficult to adapt due to defects like concrete deterioration and insufficient floor strength. To solve this problem, a composite support system of “high-strength steel mesh shell + shotcrete + bolt” was designed. Taking the roadway of Dingji Coal Mine as the engineering background, numerical simulation (ABAQUS) and similar model tests were conducted to study its bearing characteristics and optimize key parameters. Through orthogonal tests of 16 working conditions, the influence degree of parameters on the support effect was determined as: burial depth of reticulated shell floor > concrete strength > longitudinal reinforcement diameter. The optimal parameter combination (burial depth 1.0 m, longitudinal reinforcement diameter 12 mm, concrete strength C20) achieved a minimum floor heave of 14.32 mm. The stress analysis shows that the roof and floor surrounding rock are mainly under compressive stress (stress concentration at the sidewall foot), while the reticulated shell steel bars mainly bear tensile stress with a peak value of 48 MPa under long-term stable service conditions; under ultimate load, the local tensile stress of the outer secondary arc reinforcement reaches the yield strength (235 MPa), forming a ‘compression-dominant, tension–compression synergy’ bearing system, forming a “compression-dominant, tension–compression synergy” bearing system. Similar model tests verified that the composite structure delays crack propagation through synergistic action, maintaining good integrity even when the arch foot stress increases from 4.4 MPa to 9.02 MPa under 0.7 MPa load. This study confirms that the optimized high-strength composite support structure effectively improves the stability of deep soft rock roadways, providing theoretical and technical support for complex geological conditions.

## Introduction

As the “ballast stone” of China’s energy security, coal mining is facing severe support challenges in deep soft rock roadways with the increase of mining depth^1^. The deep geological environment has typical characteristics of “high erosion, high ground temperature, high osmotic pressure, high ground pressure, and strong disturbance”, leading to severe deformation of roadway surrounding rock and easy failure of support structures^2–4^. During long-term service, roadways continuously bear surrounding rock pressure and self-weight, resulting in cumulative deformation. With the depletion of shallow resources, deep mining has become an inevitable trend^5,6^. However, the increase in mining depth and intensity has caused more roadway surrounding rocks to show soft rock characteristics, and problems such as large deformation and floor heave have become increasingly prominent^7^. These problems not only threaten coal mine safety but also increase roadway maintenance costs, becoming a key bottleneck restricting coal mining^8–11^. Floor heave and large deformation of two sidewalls are the main failure forms of soft rock roadways. In particular, floor heave is difficult to control due to its special location, making it the top priority of surrounding rock control^12^.

This paper takes the deep soft rock roadway of Dingji Coal Mine (located in Huainan, Anhui Province, China) as the engineering background. Dingji Coal Mine is a typical deep mining mine with a mining depth exceeding 800 m, and the test roadway is located at the 910-level track drift with an absolute floor elevation of − 927.3 m. The mine area is characterized by complex geological conditions: the stratum sequence includes Cambrian, Ordovician, Carboniferous, Permian and Cenozoic formations, with the roadway mainly passing through mudstone, siltstone and fine sandstone layers. The surrounding rock has low strength and strong rheological characteristics. Affected by the “five-high” environment, the mine’s deep roadways face prominent engineering problems: (1) High ground temperature accelerates the hydration degradation of concrete and the corrosion of steel bars; (2) The groundwater contains high concentrations of Ca^2+^, Mg^2+^, Cl^−^ and SO₄^2−^ ions, which cause serious sulfate erosion to the support structure; (3) High ground pressure leads to excessive fragmentation of surrounding rock and obvious floor heave, with the maximum floor heave of traditional supported roadways exceeding 30 mm; (4) Strong mining disturbance causes repeated redistribution of surrounding rock stress, resulting in frequent damage to the traditional support structure. At present, the mine’s roadway maintenance mainly relies on frequent repair and reinforcement, with a short maintenance cycle and high maintenance cost , which seriously restricts the safe and efficient mining of the mine. Therefore, a new support system is urgently needed to solve the problem of stability control of deep soft rock roadways in Dingji Coal Mine.

Since the mechanism of floor heave varies with different roadway working conditions, it is necessary to select different support methods according to on-site conditions to effectively control roadway floor heave^13^. At present, scholars have found through theoretical, on-site, and numerical simulation studies based on roadway deformation mechanisms and on-site working conditions that existing supports are insufficient in supporting soft rock roadways under high-stress complex environments, resulting in excessive roadway deformation. Relevant studies have shown that the excessive deformation of roadways is due to the failure of bolts or anchor cables in the support, which makes them lose their supporting effect. Therefore, they proposed the concept of composite support^14,15^. Existing composite supports mostly focus on the mechanical synergy between bolts and anchor cables^16,17^ or the performance optimization of a single material, but there is insufficient research on the tension–compression synergy bearing mechanism of the “reticulated shell-concrete-bolt” ternary structure under high stress. Meanwhile, there are few studies on the temporal and spatial matching between the “rheological characteristics” of soft rock and the “flexible deformation” of support structures, making it difficult to adapt to the dynamic evolution of deep non-uniform stress fields. Some scholars have designed special support methods according to surrounding rock characteristics, mining influence, and structural composition, revealed the deformation mechanism of soft rock roadways, and proposed support-modification-pressure yielding control methods, which have achieved good performance in on-site applications^18,19^. Sakhno^20^ proposed anti-shear pile control technology and verified its economy and effectiveness through numerical simulation. Zhai^21^ proposed the DS-IBA support strategy for water-rich soft rock roadways. Feng^22^ studied the mechanism of floor heave failure in high-speed railway tunnels and proposed effective treatment measures. Xu^23^ developed a new type of high-performance grouting material to control floor heave in broken soft rock. Wang^24^ compared the effects of four support schemes through numerical simulation. Liu^25^, proposed a comprehensive support scheme of “floor grouting + floor bolt + sealing material”.

Despite significant progress in research on soft rock deformation control, many challenges remain^26,27^. First, the diversity of soft rock composition and structure leads to complex floor heave mechanisms, and existing studies are difficult to fully cover^28,29^. Second, with the development of underground engineering, it is necessary to combine new technologies, new materials, and new methods to promote in-depth research^30–32^. In addition, more empirical studies on soft rock deformation under different geological conditions and engineering backgrounds are needed to improve the existing theoretical system^33,34^. Therefore, developing high-strength composite support structures suitable for deep complex environments has become an urgent task^35,36^. This paper first establishes a three-dimensional model using ABAQUS^37,38^ and optimizes key support parameters through orthogonal tests^39^. Then, a physical model is constructed based on similarity theory, and the numerical results are verified using a large-scale surrounding rock-support coupling test system, finally forming a complete research chain of “parameter optimization-mechanism revelation-engineering verification”.

This paper aims to address three core issues: first, to clarify the influence weights of parameters such as burial depth of high-strength reticulated shells and diameter of longitudinal reinforcement on support effectiveness; second, to reveal the bearing mechanism of the “reticulated shell-concrete-bolt” system characterized by “compression-dominant, tension–compression synergy”; third, to elucidate the instability evolution path of the support structure under non-uniform stress fields. The research findings can not only directly guide the optimization of roadway support schemes in Dingji Coal Mine but also provide a replicable technical framework for support engineering of soft rock roadways under other deep and complex geological conditions through the proposed orthogonal parameter optimization method and the “tension–compression synergy” design concept. Furthermore, discussions on potential challenges in large-scale implementation will offer practical guidance for engineering applications in similar projects.

## Design of high-strength steel mesh shell lining structure

**Fig. 1:**
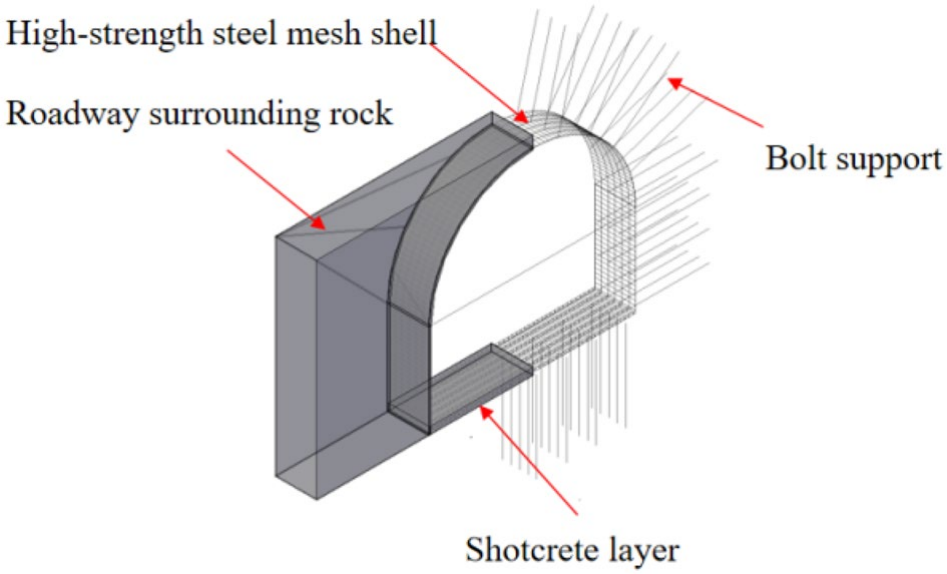
High-strength steel mesh shell composite support roadway.

Support for roadways in weak surrounding rock needs to balance structural stability and deformation coordination. Traditional support technologies are difficult to adapt to complex conditions such as “high erosion, high ground pressure, and strong disturbance” in soft rock roadways due to insufficient structural continuity and low load transfer efficiency^40–42^. Therefore, a composite support system of “high-strength steel mesh shell + shotcrete + bolt” is developed. It realizes the synergistic improvement of shear and tensile properties through a three-dimensional space truss structure, and absorbs surrounding rock energy using a flexible deformation mechanism, forming a “reticulated shell-concrete-bolt” ternary coupling mechanism to adapt to the rheological properties of weak surrounding rock, as shown in Fig. 1.

**Fig. 2:**
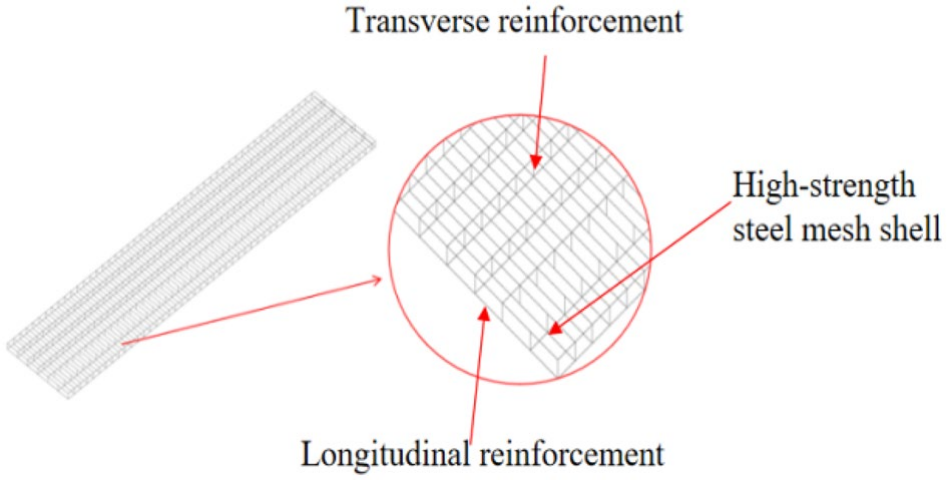
Steel mesh shell structure.

The core design of the system includes five parts:

1. Straight wall structure: It bears the vertical load from the top, realizes uniform stress transfer through rigid support, and improves the self-bearing capacity of surrounding rock. It plays a decisive role in maintaining the integrity of the roadway space in medium-hard rock formations.
2. Arch load-bearing system: The semicircular arch structure converts lateral ground pressure into circumferential stress, inhibits the expansion of the plastic zone of surrounding rock through circumferential constraints, and the rise-span ratio directly affects the safety reserve coefficient.
3. Steel mesh shell system: It forms a three-dimensional load-bearing structure in synergy with shotcrete, optimizes tension–compression stress using material anisotropy, regulates the interaction between surrounding rock and support through node deformation coordination, and realizes energy dissipation in rheological formations, as shown in Fig. 2.
4. Concrete bearing layer: As the main pressure-bearing interface, its rheological properties are temporally and spatially synergistic with surrounding rock deformation, forming a compression-tension complementary mechanism with the reticulated shell. Its self-healing function can maintain interface strength under alternating humidity environments, ensuring full-life performance.
5. Anchoring reinforcement system: Full-length bonded bolts establish a mechanical connection between deep surrounding rock and support, improve the stress state through prestress diffusion, enhance the shear strength of structural planes in weak interlayer areas, and lock potential sliding surfaces.

## Numerical simulation and scheme optimization of high-strength composite support structure

### Model establishment and parameter setting

**Fig. 3:**
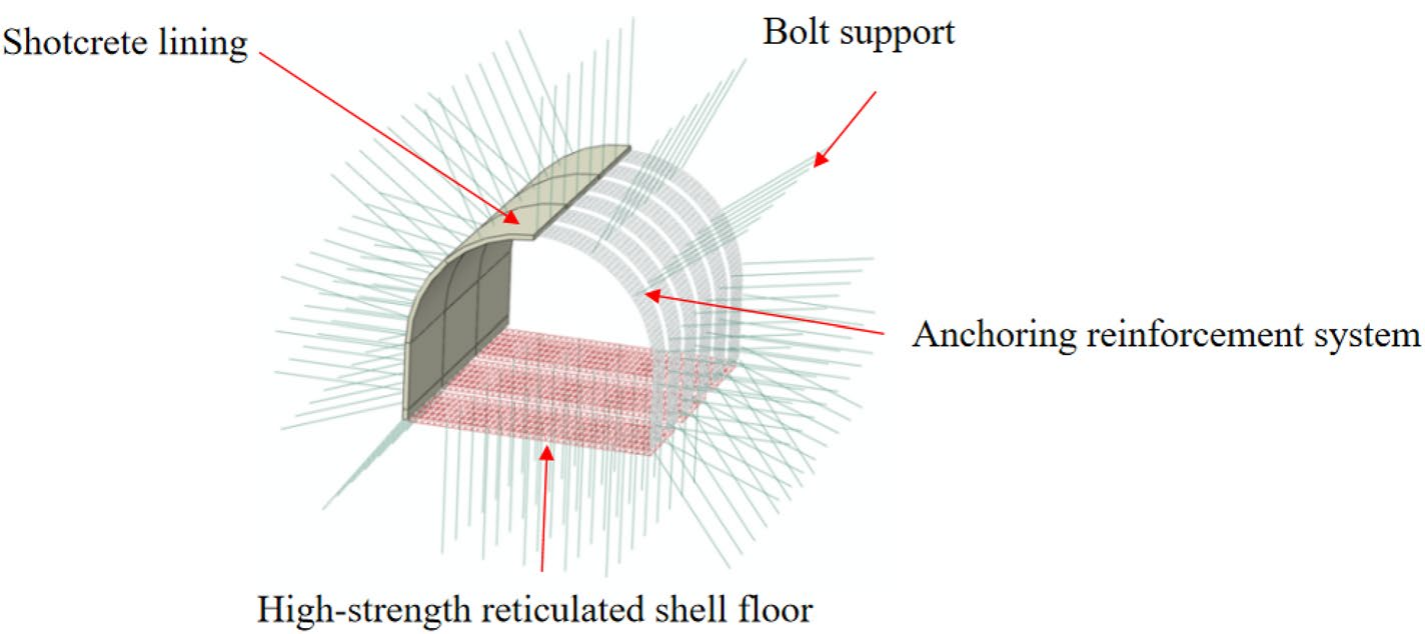
Schematic diagram of the internal structure of the roadway lining model.

Based on the engineering background of soft rock roadways in Dingji Coal Mine, a three-dimensional numerical model was established using ABAQUS, with dimensions of 50 m × 50 m × 6 m, including coal seams, mudstone, siltstone, and fine sandstone. The roadway has a straight-wall semicircular arch section with a net width of 5.4 m and a net height of 4.3 m (arch height 2.7 m, straight wall height 1.6 m). The support structure includes a high-strength reticulated shell floor and a shotcrete layer (see Fig. 3).

The surrounding rock adopts the Mohr–Coulomb constitutive model, the concrete in the support structure adopts a linear elastic model, and the steel mesh shell adopts an ideal elastoplastic model. For the numerical model, key assumptions and simplifications include that surrounding rock strata are homogeneous and isotropic within each layer with adjacent strata perfectly bonded, the support structure is fully bonded to the surrounding rock, and roadway excavation is simplified as one-step excavation to focus on long-term stability; boundary conditions involve displacement constraints, bottom surface fixed vertically, four side surfaces fixed horizontally, X-boundaries = 0, Y-boundaries = 0, top surface free with overburden pressure of 20 MPa calculated as $\sigma = \gamma H$ where *γ* = 25 kN/m^3^and *H* = 800 m) and stress constraints (horizontal in-situ stress applied with a lateral pressure coefficient of 1.0 consistent with field measurements, no pore water pressure as the stratum is dry verified by field surveys); material models are defined as follows: surrounding rock adopts the Mohr–Coulomb constitutive model to capture shear failure with parameters from laboratory core tests, concrete uses a linear elastic model as stress is less than yield strength in the optimal scheme, and the steel mesh shell/bolts adopt an ideal elastoplastic model based on the von Mises criterion with steel remaining elastic in most service periods.

The key physical and mechanical parameters are shown in Tables 1 and 2.

**Table 1:** Physical and mechanical parameters of surrounding rock materials.

Rock stratum	Density/kg m^−3^	Elastic modulus/GPa	Poisson’s ratio	Yield stress/MPa	Internal friction angle/°
Coal seam	1420	0.73	0.32	2.3	37.7
Mudstone	2771	2.74	0.14	39.2	50.7
Siltstone	2709	6.35	0.20	18.8	52.4
Fine sandstone	2977	8.41	0.16	14.0	59.1

**Table 2:** Material parameters of support structure.

Material	Density/kg m^−3^	Elastic modulus/GPa	Poisson’s ratio	Yield stress/MPa
Concrete	2400	30	0.2	30
Steel bar	1800	200	0.3	235

### Orthogonal test design

The burial depth of the high-strength reticulated shell floor (A), the diameter of longitudinal reinforcement (B), and concrete strength (C) were selected as influencing factors, with 4 levels set for each factor, as shown in Table 3. A total of 16 groups of tests were designed using the L_16_ (4^5^) orthogonal table, and the floor heave was used as the evaluation index to optimize the support parameters.

**Table 3:** Factor level table of simulation schemes.

Level	Floor burial depth/m	Diameter of longitudinal reinforcement in reticulated shell/mm	Lining concrete strength
1	0.6	6	C15
2	0.8	8	C20
3	1.0	10	C25
4	1.2	12	C30

### Deformation and stress laws of support system

1. Deformation characteristics.

**Fig. 4:**
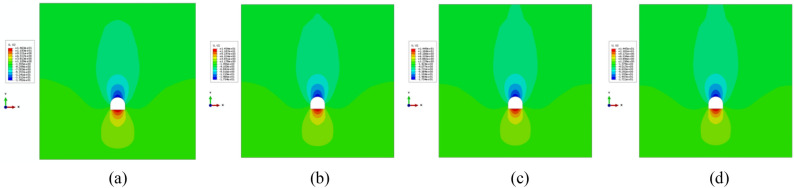
Distribution of vertical displacement field of roadway surrounding rock with different burial depths.

**Fig. 5:**
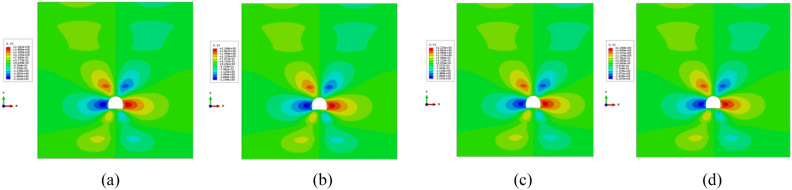
Distribution of horizontal displacement field of roadway surrounding rock with different burial depths.

The vertical displacement of surrounding rock spreads to the deep in a “fish scale” pattern, and the displacement gradually decreases with increasing distance from the roadway surface. As shown in Fig. 4, under the working condition of a burial depth of 0.6 m, the maximum displacement of the roof is 17.82 mm, and the floor heave is 14.63 mm; when the burial depth increases to 1.0 m, the roof displacement decreases to 17.34 mm, and the floor heave reduces to 14.49 mm. As shown in Fig. 5, the horizontal displacement shows a “butterfly” distribution, and the displacement in the foot of the sidewall is the most sensitive, decreasing from 2.26  to 2.21 mm.

**Fig. 6:**
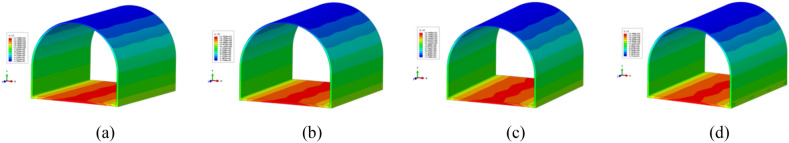
Distribution of vertical displacement field of lining structure under different burial depths.

**Fig. 7:**
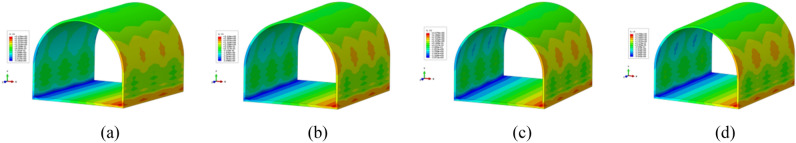
Distribution of horizontal displacement field of lining structure under different burial depths.

**Fig. 8:**
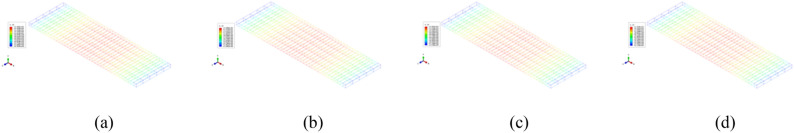
Vertical displacement nephogram of high-strength floor reticulated shell under different burial depths.

**Fig. 9:**
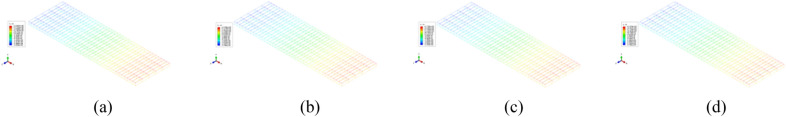
Horizontal displacement nephogram of high-strength floor reticulated shell under different burial depths.

As shown in Figs. 6 and 7, the lining displacement is concentrated in the arch crown and floor, and the attenuation amplitude of floor displacement is greater than that of the arch crown. As shown in Figs. 8 and 9, the deformation of the high-strength floor reticulated shell is symmetrically distributed, with the maximum vertical displacement in the middle, decreasing from 14.42 mm to 14.19 mm, and the horizontal displacement at the end slightly increasing with the increase of burial depth.

(2) Stress characteristics.

**Fig. 10:**
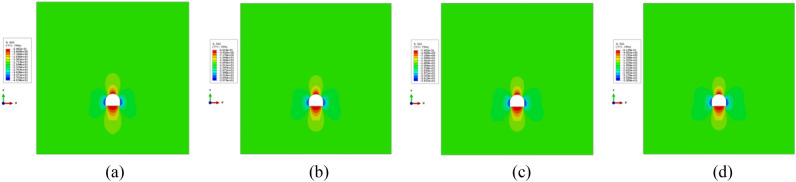
Contour plot of support surrounding rock stress at different burying depths.

**Fig. 11:**
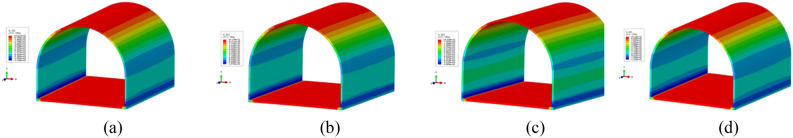
Vertical stress nephogram of lining structure under different burial depths.

As shown in Fig. 10, the surrounding rock is mainly under compressive stress, and there is always stress concentration at the foot of the sidewall, decreasing from 40.34 MPa to 39.08 MPa, because this part is a geometric mutation of the section with concentrated plastic zones. As shown in Fig. 11, the lining stress is concentrated at the connection between the foot of the sidewall and the floor. At a burial depth of 1.0 m, the peak compressive stress is the lowest at 99.95 MPa. Excessive burial depth leads to a sudden increase in stress due to the decrease in structural synergy.

**Fig. 12:**
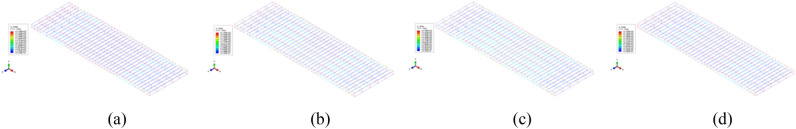
Mises stress distribution characteristics of steel mesh shell under different burial depths.

As shown in Fig. 12, the high-strength floor latticed shell reinforcement primarily bears tensile stress, with the outer secondary arc reinforcement exhibiting significant response and reaching a peak value of 235 MPa. Note: The peak tensile stress of 235 MPa is the local yield stress of the outer secondary arc reinforcement under ultimate load, which is consistent with the yield strength of the steel bar (Table 2). Under long-term stable service conditions (optimal parameter combination), the overall peak tensile stress of the reticulated shell steel bars is 48 MPa, reflecting the conventional stress-bearing level of the structure during normal operation. directly undertaking the tension induced by lateral deformation. Local yielding occurs at the ends, necessitating structural optimization to enhance anchorage strength. The tensile stress in the latticed shell and the compressive stress in the lining complement each other, forming a composite bearing system characterized by “compression-dominated, tension–compression synergy”.

### Determination of optimal scheme

To determine the optimal support parameters, range and variance analysis methods were used, with floor heave as the evaluation index, to analyze the influence laws of the burial depth of the high-strength floor (A), the diameter of longitudinal reinforcement in the reticulated shell (B), and concrete strength (C).

The range analysis results show (see Table 4) that the influence degree of each factor on floor heave is: A > C > B, with range values R of 0.16, 0.06, and 0.038, respectively. The optimal level combination is A_3_B_4_C_2_ (burial depth 1.0 m, longitudinal reinforcement diameter 12 mm, concrete strength C20), corresponding to the minimum floor heave of 14.32 mm in working condition 12.

**Table 4:** Key results of range analysis.

Factor	Level 1	Level 2	Level 3	Level 4	Range value *R*	Influence order
A (burial depth/m)	14.63	14.55	14.47	14.47	0.16	1
B (longitudinal reinforcement diameter/mm)	14.53	14.53	14.55	14.52	0.038	3
C (concrete strength)	14.50	14.56	14.56	14.51	0.06	2

The data in the table are the average floor heave of each level (mm).

Variance analysis further verified that the influence of burial depth on floor heave is extremely significant (*F* = 16.602 > *F*_0.01_), the influence of concrete strength is weak, and the diameter of longitudinal reinforcement has no significant influence, which is consistent with the range analysis.

Supplementary statistical analysis based on the F-distribution shows (see Table 5) that the p-value of burial depth (A) is 0.002 (*p* < 0.01), indicating an extremely significant effect on floor heave; the p-value of concrete strength (C) is 0.128 (*p* > 0.05), representing a weak effect; and the p-value of longitudinal reinforcement diameter (B) is 0.586 (*p* > 0.05), showing no significant effect. Additionally, the 95% confidence interval of floor heave for the optimal scheme (A_3_B_4_C_2_) is [14.01 mm, 14.63 mm], which reflects the high stability of its control effect.

**Table 5:** Analysis of variance table.

Factor	Sum of squares	Degrees of freedom	Mean square	*F*	p-value	Significance
A (burial depth/m)	0.069	3	0.023	16.602	0.002	**
B (longitudinal reinforcement diameter/mm)	0.003	3	0.001	0.735	0.586	–
C (concrete strength)	0.013	3	0.004	3.217	0.128	–
Error *e*	0.152	6	0.025	–	–	–

Considering engineering economy and control effect, the final optimal scheme is A_3_B_4_C_2_ (burial depth 1.0 m, longitudinal reinforcement diameter 12 mm, concrete strength C20).

## Design of similar model test

### Model loading system

**Fig. 13:**
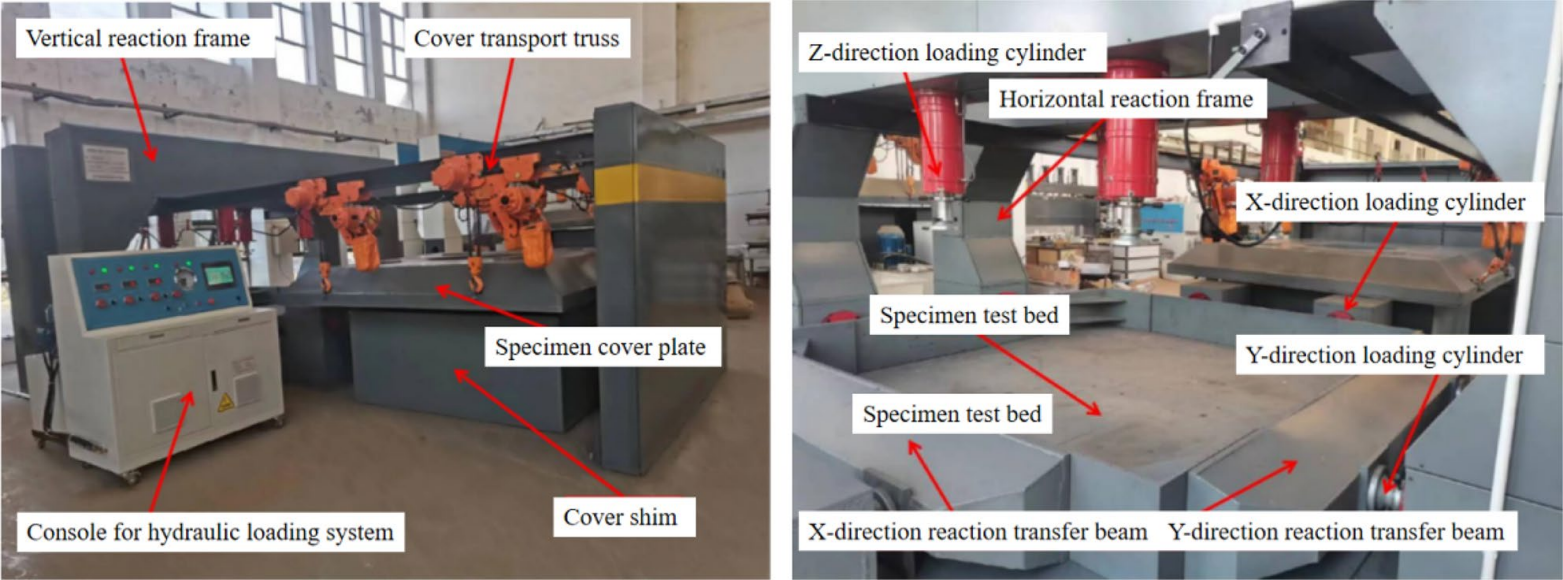
Model test system for coupling effect between large-scale surrounding rock and lining structure.

Building upon numerical simulations and orthogonal test results, this study identified the optimal parameter combination for the high-strength steel mesh shell straight-wall semicircular arch lining structure. A large-scale surrounding rock-lining structure coupling test system, fabricated by Sichuan Pusheng Technology Co., Ltd. (China), was employed for experimental validation, as illustrated in Fig. 13.

Comprising a main frame, a hydraulic drive module, and an intelligent control unit, the test system exhibits a maximum axial bearing capacity of 2400 kN. The model chamber, with internal dimensions of 3200 mm × 3200 mm, is capable of accommodating specimens sized 2500 mm × 2500 mm × 300 mm, which fulfills the requirements of scaled model tests for kilometer-deep roadways.

Notably, the system supports independent or synchronous loading along the X and Y directions, while a passive constraint mode is adopted for the Z direction. The hydraulic drive module incorporates a digital closed-loop control architecture, integrating high-precision proportional valves and servo cylinders that operate synergistically. With a loading error margin of ≤  ± 1%, the system implements real-time dynamic compensation for pressure fluctuations based on the proportional-integral-derivative (PID) algorithm, ensuring long-term stable output of triaxial loads.

This advanced test system enables accurate simulation of surrounding rock deformation processes under the coupling effects of varying burial depths and horizontal in-situ stresses, while simultaneously outputting real-time data on the internal forces of the support structure and the evolutionary characteristics of surrounding rock stresses.

### Similarity criteria and model parameters

Taking the straight-wall semicircular arch roadway in the safety reconstruction and second-level extension project of Dingji Coal Mine as the prototype, its inner and outer diameters of the section are 5.4 m and 5.6 m, respectively, with a wall thickness of 0.1 m and a straight wall height of 1.6 m. Combined with the constraint conditions of the three-dimensional similar model test device, the geometric similarity constant *C*_*L*_ = 20 was set. According to the bulk density proportional coefficient derived from formula (1), based on the established geometric similarity ratio *C*_*L*_ = 20, the stress similarity ratio $C_{\sigma }$ = 30 was obtained. Through systematic parameter conversion calculation, the similarity ratio of mechanical parameters of model materials was obtained, where $C_{\smallint }$ = $C_{\mu }$ = 1, $C_{\sigma }$ = $C_{c}$ = $C_{\phi }$ = 30.

1 $$C_{s} = \frac{{\sigma_{H} }}{{\sigma_{M} }} = C_{I} \times C_{r} = C_{I} \times \frac{{\gamma_{H} }}{{\gamma_{M} }}$$

In the formula, $C_{\sigma }$ is the stress similarity ratio; $C_{\gamma }$ is the stress similarity constant; $\sigma_{H}$ and $\sigma_{M}$ are the prototype and model stresses; $\gamma_{H}$ and $\gamma_{M}$ are the prototype and model bulk densities.

**Fig. 14:**
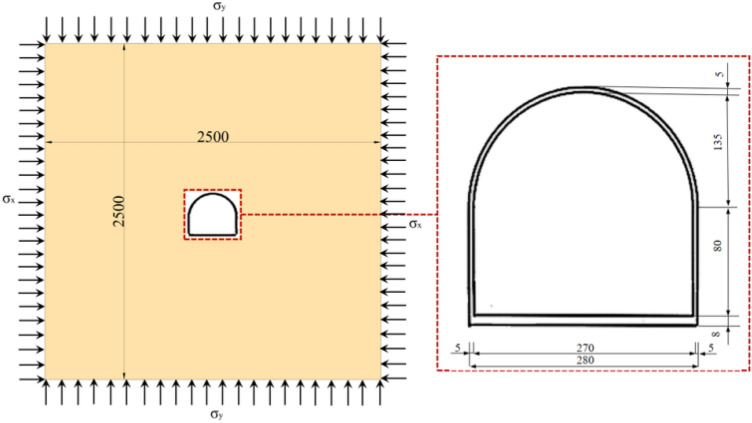
High-strength steel mesh shell straight-wall semicircular arch support scheme.

Through theoretical analysis and parameter optimization, the straight-wall semicircular arch steel mesh shell lining support scheme shown in Fig. 14 was finally formed. Based on the measured data of rock mechanics parameters and similarity criteria, the surrounding rock similar material uses Huaihe medium sand as aggregate and gypsum as cementitious component. The optimal ratio determined through orthogonal proportioning tests is 8:2. This material has a bulk density of 1.6 kN/m^3^, a load of 1.0 MPa, a Poisson’s ratio of 0.25, a cohesion of 0.03 MPa, and an internal friction angle of 52.4°, which can accurately reproduce the physical and mechanical characteristics of natural surrounding rock, as shown in Table 6.

**Table 6:** Mechanical parameters of surrounding rock similar materials.

Mechanical parameter	Bulk density/kN m^−3^	Load /MPa	Poisson’s ratio	Cohesion/MPa	Internal friction angle/°	Ratio
Prototype	2.4	30.5	0.25	0.91	52.4	8:2
Model	1.6	1.0	0.25	0.03	52.4	
Similarity ratio	1.5	30	1	30	1	

**Fig. 15:**
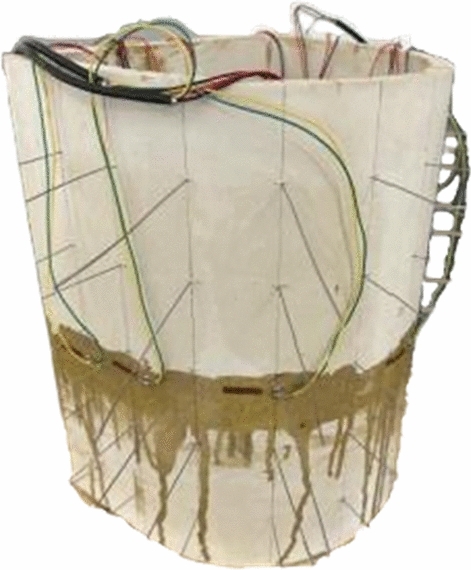
Macroscopic morphology of the specimen.

The lining material adopts gypsum-based composite material, and the mass ratio of gypsum to water is determined as 1:1 based on orthogonal test results, with a nominal compressive strength of 0.8 MPa and an elastic modulus of 1.0 GPa. The arch part is constructed with a 5 mm equal-thickness spray layer, and the foundation transition zone is optimized for bearing gradient through an 8 mm thickened structure. Following the principle of geometric similarity priority, the steel mesh components are constructed with φ0.3 mm fine wires, and the floor shell is configured with φ0.6 mm steel wires for structural simulation. The bolts are made of φ1.0 mm high-strength steel wires and a 3:1 resin-hardener composite system, arranged at a standardized spacing of 35 × 40 mm. The macroscopic morphology of the formed specimen is shown in Fig. 15, verifying the rationality and feasibility of the model structure.

**Fig. 16:**
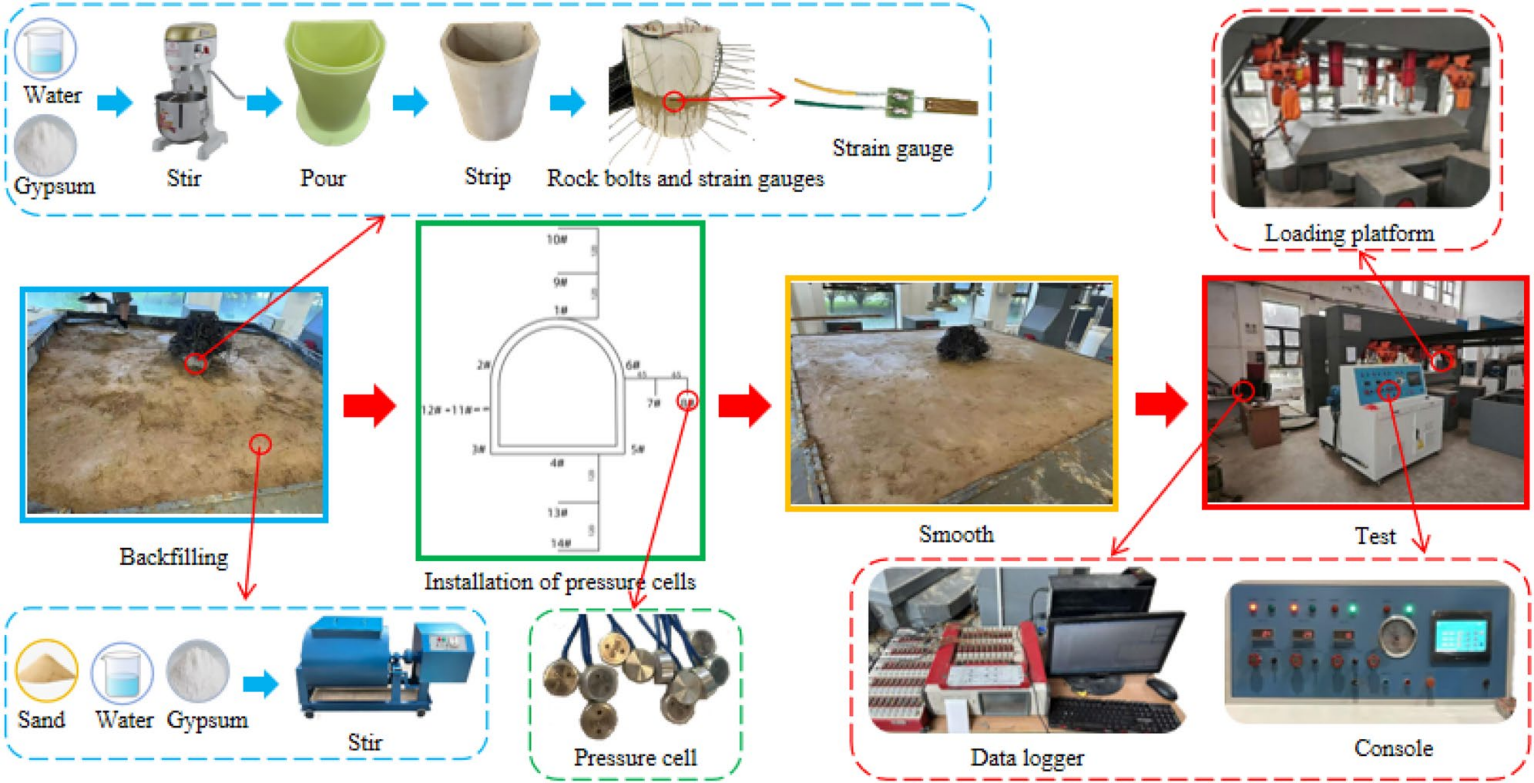
Key process flow of model preparation.

Based on multi-source error control theory, a standardized control system for process parameters and environmental conditions needs to be established before model preparation. This system achieves source control of system errors by maintaining the homology of material preparation, storage, and reference test conditions. The specific implementation process is shown in Fig. 16.

### Loading and monitoring scheme

In the design of the test load transfer mechanism and loading path, full consideration is given to the significant regulatory effect of the lateral pressure coefficient on the stress redistribution of surrounding rock and the internal force transmission of the support structure. A multi-stage loading mode was adopted in the test, as detailed in Table 7.

**Table 7:** Multi-directional stress loading parameter configuration table.

Test stage	Simulated depth/m	Y-direction load/MPa	Lateral pressure coefficient	X-direction load/MPa				
Stage 1	300	0.3	0.8 0.9 1.0 1.1 1.2	0.24	0.27	0.30	0.33	0.36
Stage 2	500	0.5		0.40	0.45	0.50	0.55	0.60
Stage 3	700	0.7		0.56	0.63	0.70	0.77	0.84
Stage 4	900	0.9		0.72	0.81	0.90	0.99	1.08

For the Y-axis load, a stepwise increment strategy was employed based on the simulation of roadway burial depth. The initial load was set at 0.3 MPa, with an increment step of 0.2 MPa, reaching a maximum of 0.9 MPa, which corresponds to simulated burial depths ranging from 300 to 900 m. For the X-axis load, it was configured within the range of 0.8–1.2 according to the lateral pressure coefficient, with an increment step of 0.1. During the test implementation, the X and Y axial loads were applied synchronously, while the Y-direction load was maintained constant at each loading stage.

The cover constraint system achieved displacement locking in the Z-direction, and the hydraulic servo system performed load tracking control to ensure loading accuracy. The test adopted a multi-stage load transfer procedure, where each stage had a loading cycle of 30 min. After the completion of load transfer in each stage, an 8-h pressure holding period was implemented. The next stage of load transfer could only be initiated after the pressure holding period was completed, ensuring the full development of surrounding rock deformation and stress adjustment.

The monitoring scheme includes lining internal force monitoring and surrounding rock stress monitoring.

**Fig. 17:**
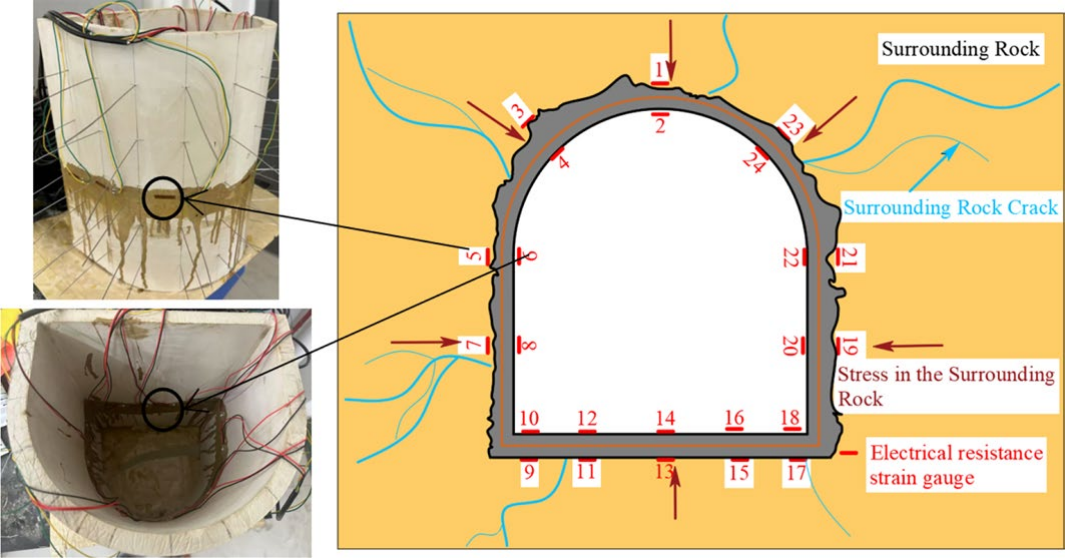
Schematic diagram of strain gauge layout.

1. Lining internal force monitoring: 24 BX120-20AA resistance strain sensors are used, configured with a 20 mm × 3 mm measuring grid structure, with a calibration sensitivity coefficient of 2.08 ± 1%. They are arranged symmetrically at equal intervals on the outer surface of the lining specimen, adopting a full circumferential coverage array configuration, with 11 standardized monitoring points, as shown in Fig. 17. Signal acquisition is realized through a TDS-630 static signal acquisition instrument. The axial force and bending moment of the lining structure can be derived from internal force parameters, and the relevant calculations are shown in formulas (2) and (3).

2 $$N = \frac{{E\left( {\varepsilon_{Inner} + \varepsilon_{Outer} } \right)bh}}{2}$$

3 $$M = \frac{{E\left( { \varepsilon _{Inner} - \varepsilon _{Outer} } \right)bh^{2} }}{12}$$

where the material elastic modulus *E* is 1.0 GPa, the specimen section width *b* represents the dimension in the unit length direction, the lining structure thickness parameter is designed differently, with 5 mm for the straight-wall semicircular arch section and 8 mm for the bottom area, $\varepsilon_{Inner}$ and $\varepsilon_{Outer}$ are the measured microstrain values of the inner and outer sides of the lining, respectively.

**Fig. 18:**
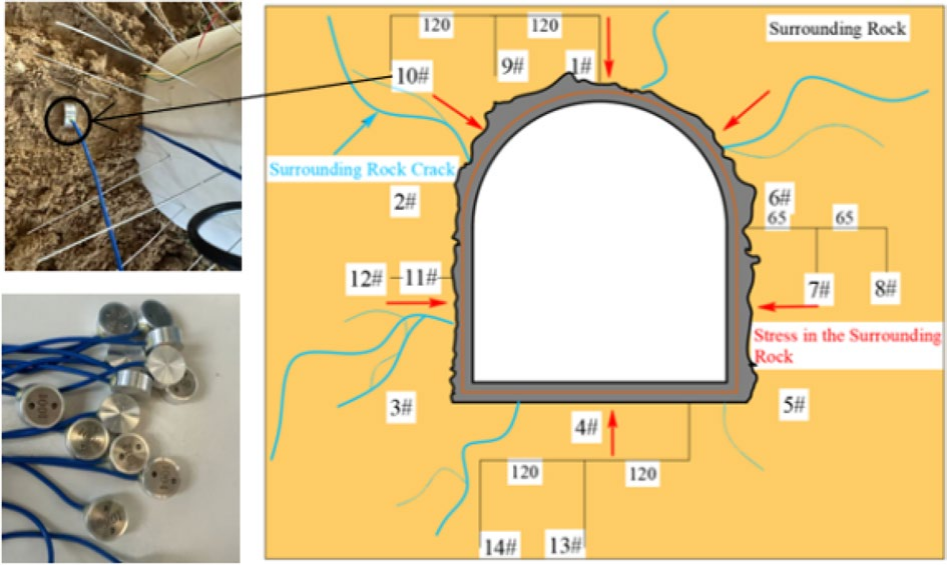
Layout of lining support pressure boxes.

(3) Surrounding rock stress monitoring: 14 LY-350 micro-strain earth pressure sensors are selected, with a 28 mm sensing surface diameter and a 1.5 MPa range design. Six groups of stress monitoring arrays numbered 1# ~ 6# are arranged at equal distances on the interface layer of the support structure. For the lateral stress conduction characteristics of the structure, four groups of stress monitoring units (numbered 7#, 8#, 11#, 12#) are symmetrically arranged in the 65 mm stress gradient zone of the two sidewalls of the support body, constructing a horizontal stress field gradient network with equal axial intervals. Meanwhile, for the key bearing area of the arch crown-base, four groups of high-sensitivity stress sensing arrays (numbered 9#, 10#, 13#, 14#) are set, and stress field reconstruction is performed with a 120 mm spatial resolution, as shown in Fig. 18.

## Analysis of similar model test results

### Analysis of lining structure failure

During the model loading test, as the Y-direction load increased to 0.9 MPa, dense longitudinal cracks propagated over the lining surface, ultimately resulting in overall structural instability and loss of support functionality. The spatial distribution characteristics of these cracks can effectively reveal the underlying failure mechanism of the lining structure, thereby providing a reliable theoretical and experimental basis for safety assessment and optimization of the support scheme.

**Fig. 19:**
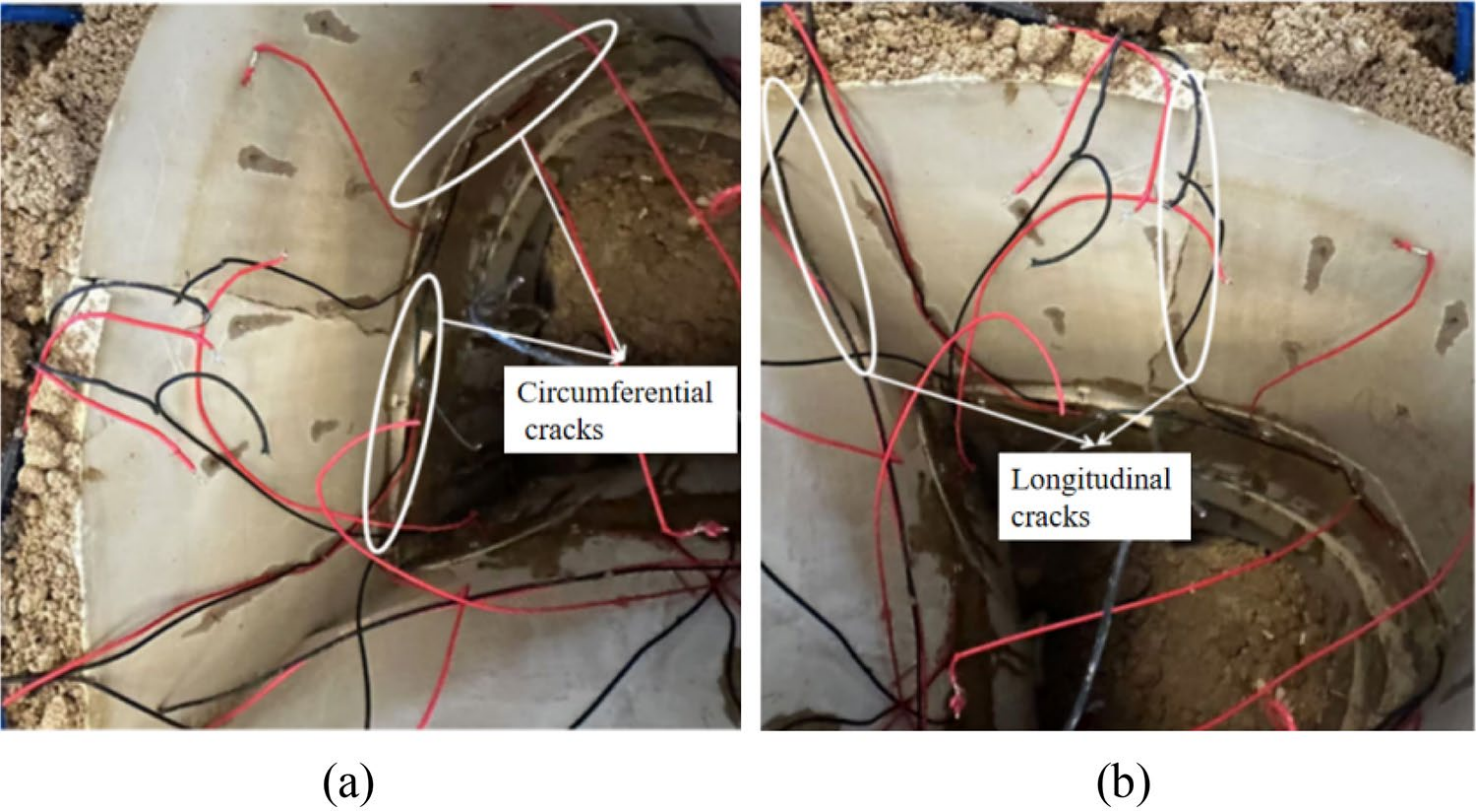
Spatial evolution characteristics of circumferential and axial cracks in lining structure.

As shown in Fig. 19, there are circumferential and axial fracture networks on the structure surface. In Fig. 19a, there is a significant circumferential crack zone in the strain gauge layout area, and a through failure interface is formed at the arch foot, indicating that a significant stress gradient is generated in the central axis area of the lining during load transfer, causing local strain energy accumulation and material fracture, destroying the continuity of the bearing interface, and aggravating structural instability through stress redistribution, ultimately leading to the degradation of support function; Fig. 19b shows that typical axial cracks develop at the arch foot and arch shoulder, and their distribution is directly related to the non-uniform high stress state, belonging to tension-shear composite failure. The geometric mutation at the arch foot and arch shoulder aggravates stress redistribution, accelerating damage in weak parts. In summary, the main cause of lining failure is progressive failure induced by local high stress concentration, accompanied by regional instability caused by stress mutation.

**Fig. 20:**
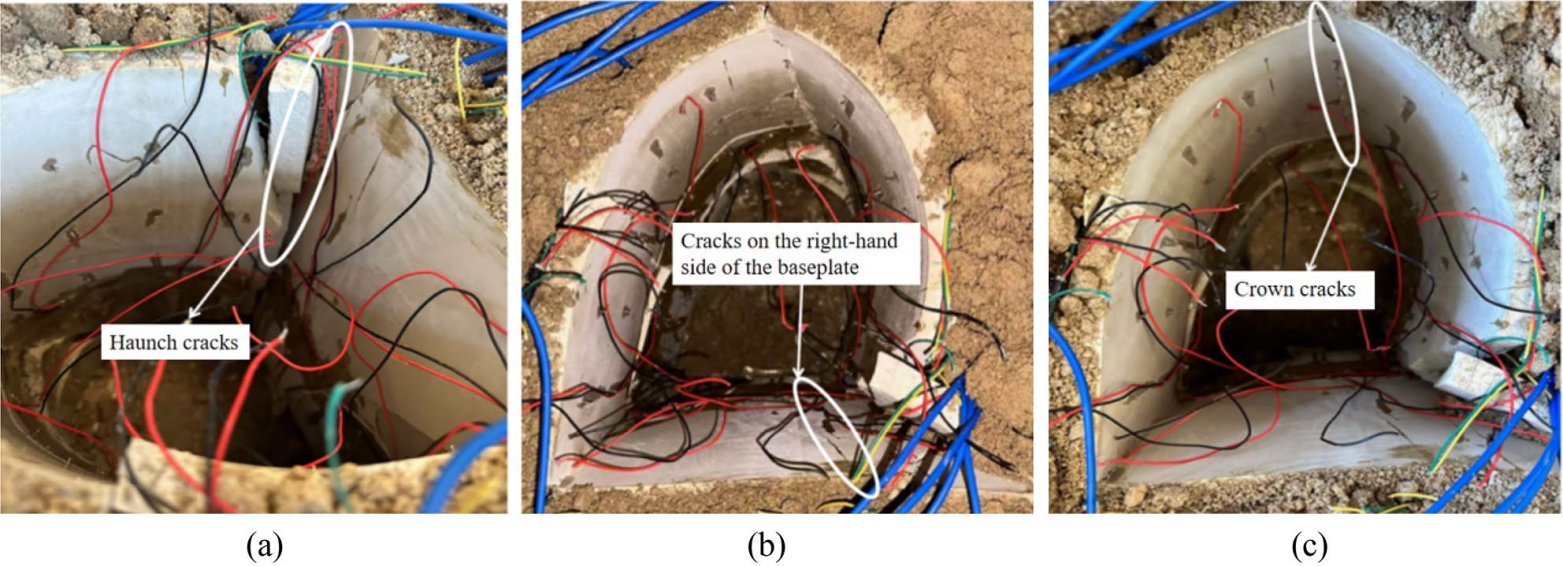
Distribution diagram of main cracks in various parts of the lining structure.

The damage distribution characteristics of each area of the lining structure are shown in Fig. 20. When the Y-direction load is 0.7 MPa, through axial cracks first appear in the arch foot area, as shown in Fig. 20a, with an opening exceeding the critical value, belonging to the main control cracks; with the increase of load, similar cracks appear in the arch crown area, as shown in Fig. 20b, with expansion rate and scale showing typical failure characteristics; during the Y-direction load holding period of 0.9 MPa, the bottom plate shows differential longitudinal damage on both sides, as shown in Fig. 20c, with the left crack opening reaching the main control threshold and the right crack being a secondary crack, both expanding inward, initiating from the inner edge without penetration, and the development depth is controllable.

Test results indicate that damage initiates at the sidewall foot during the early loading stage, with the crack propagation rate and scale being significantly higher than those in other regions. The underlying mechanism lies in the fact that this area serves as the transition interface between the arch and the straight wall, where geometric discontinuity induces stress concentration—this constitutes the primary cause of progressive material failure. A through crack forms at the arch foot when the load reaches 0.7 MPa, and evolves into the dominant failure mode at 0.9 MPa.

Accordingly, it is recommended to optimize the structural parameters of the transition zone, implement real-time strain monitoring, and enhance the interface bonding quality to suppress abnormal stress accumulation. In contrast, the floor cracks exhibit symmetrical and non-penetrating characteristics, demonstrating that the straight wall-semicircular arch structure possesses excellent bearing capacity against the surrounding rock pressure of the roadway floor.

This controllable cracking mode enables effective regulation of floor deformation and inhibition of floor heave. Furthermore, the geometric stiffness of the straight wall section mitigates the peak stress of the floor through stress homogenization, thereby enhancing the overall deformation resistance of the structure and extending its service life.

### Surrounding rock stress distribution characteristics under different lateral pressure coefficients

In accordance with the test design protocol, a multi-channel data acquisition system was employed to conduct real-time monitoring of the evolutionary characteristics of the surrounding rock stress field at each loading stage of the test roadway. The primary focus was to investigate the regulatory mechanism of the lateral pressure coefficient on the mechanical response of the surrounding rock.

Four characteristic stress levels corresponding to Y-axis loading gradients were selected as analytical benchmarks, namely 0.3 MPa (equivalent to 9 MPa for the prototype), 0.5 MPa (equivalent to 15 MPa for the prototype), 0.7 MPa (equivalent to 21 MPa for the prototype), and 0.9 MPa (equivalent to 27 MPa for the prototype). The measured data were converted using similarity criteria to reconstruct the evolutionary patterns of the surrounding rock contact stress field and the vertical stress of the rock mass within the lateral pressure coefficient range of 0.8–1.2 for the prototype roadway.

Given the geometric symmetry of the specimen and the bidirectional symmetry of the loading system, data from the sensor array on the right side of the central axis were selected for mechanical parameter interpretation to enhance analytical efficiency.

1. Characteristics of surrounding rock pressure distribution on the lining surface.

**Fig. 21:**
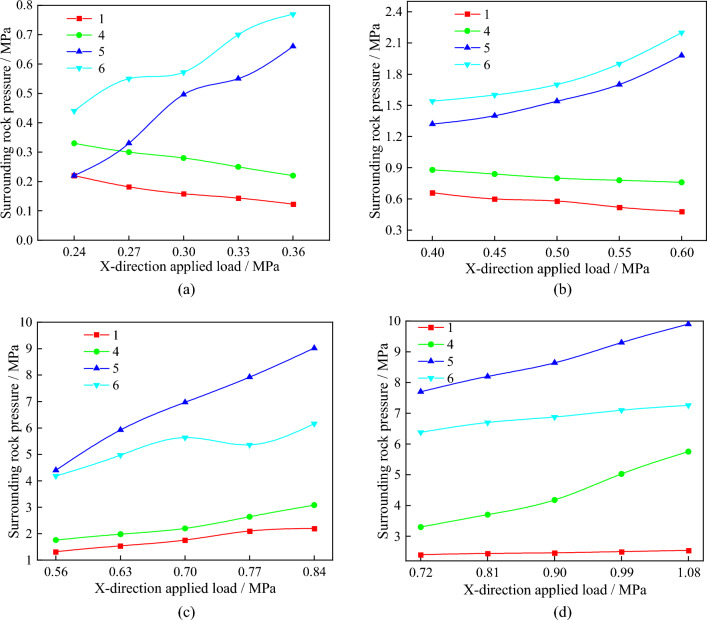
Curves of surrounding rock pressure changes under different lateral pressure coefficients.

As shown in Fig. 21. At a Y-axis load of 0.3 MPa, the contact stress of the roof (measuring point 1) and floor (measuring point 4) decreases slightly with the increase of lateral pressure coefficient; the pressure at the arch foot (measuring point 5) and arch shoulder (measuring point 6) continues to rise. At a Y-axis load of 0.5 MPa, the roof and floor stress still decrease, while the pressure increment at the arch foot and arch shoulder is more significant and increases with the lateral pressure coefficient. At a Y-axis load of 0.7 MPa, the roof and floor stress turns to a slow increase, and the arch foot stress jumps from 4.4  to 9.02 MPa. At a Y-axis load of 0.9 MPa, the top shoulder stress tends to be stable, and the floor continues to be pressurized. Although the lining is damaged, the floor still bears the load.

The test shows that under low Y-direction load, the enhancement of horizontal lateral pressure weakens the roof and floor stress; under high Y-direction load, the horizontal lateral pressure dominates the sidewall stress, showing a linear increment mode.

(2) Characteristics of vertical stress distribution in surrounding rock outside the lining.

**Fig. 22:**
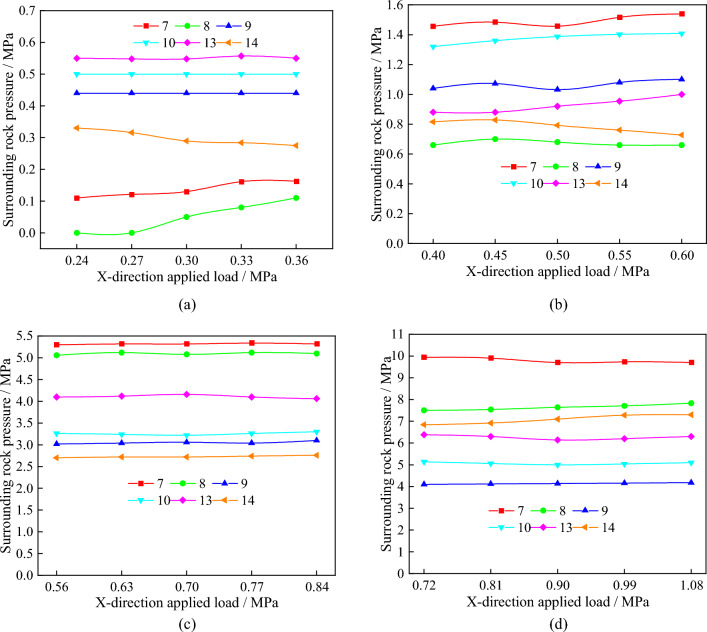
Curves of surrounding rock vertical stress changes under different lateral pressure coefficients.

The vertical stress distribution characteristics are shown in Fig. 22. Figure 22a shows that under the Y-axis load of 0.3 MPa, the vertical stress of the surrounding rock outside the two sidewalls (measuring points 7, 8) increases gradually with the increase of lateral pressure coefficient, while the stress value of the surrounding rock outside the roof (measuring points 9, 10) remains stable, which may be related to the fact that the surrounding rock material has not fully entered the bearing state; no significant stress fluctuation is observed in the surrounding rock outside the floor (measuring points 13, 14). From the comparison of Fig. 22b–d, when the Y-axis load is increased to 0.9 MPa, the vertical stress at each measuring point has no obvious response to the change of X-direction load, and the maximum vertical stress value is recorded at measuring point 7. The test shows that the horizontal lateral pressure has a limited regulatory effect on the vertical stress of surrounding rock, and the vertical stress will only increase significantly when the Y-direction load reaches a specific critical threshold. This phenomenon may be due to the adaptability of the internal stress transfer mechanism of surrounding rock, enabling it to maintain the relative balance of the vertical stress field under multi-directional loads.

### Surrounding rock stress distribution characteristics under the same lateral pressure coefficient

To grasp the stress distribution of surrounding rock mass, it is necessary to investigate the coupling effect of lateral pressure coefficient and burial depth. Through Y-axis stepwise loading, the response of surrounding rock stress field under different burial depths is reproduced, focusing on revealing the influence mechanism of Y-direction load enhancement on the support interface pressure and deep stress field when the lateral pressure coefficients are 0.8, 1.0, and 1.2.

1. Characteristics of surrounding rock pressure distribution on the lining surface.

**Fig. 23:**
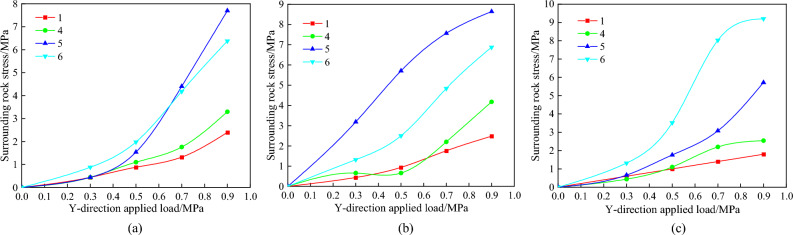
Evolution law of surrounding rock contact pressure under constant lateral pressure conditions.

As shown in Fig. 23, under constant lateral pressure, the surrounding rock pressure increases sharply with the increase of lateral pressure coefficient. After the Y-axis load exceeds 0.3 MPa, the stress gradient increases exponentially, and the stress at the foot and shoulder of the sidewall fluctuates significantly, being sensitive to lateral pressure parameters. The normal stress at the arch crown changes quasi-linearly with the Y-direction load. It stabilizes around 0.65 MPa when the lateral pressure coefficient is less than 1.0, and increases non-linearly after 0.7 MPa. The base response is similar, maintaining bearing capacity despite micro-cracks at 0.5 ~ 0.7 MPa. The sidewall stress increases linearly with the Y-direction load, with a peak value of 9.25 MPa; when the lateral pressure coefficient is 1.0, the stress is abnormally enhanced in the 0.5 ~ 0.7 MPa range, possibly entering the plastic stage. The arch shoulder forms a high stress of 9.92 MPa due to increased lateral pressure, showing three-dimensional concentration.

In summary, the straight-wall semicircular arch structure has a good effect on controlling the roof and floor pressure, but the stress at the foot of the sidewall increases sharply with burial depth, requiring local reinforcement to ensure synergistic bearing.

(2) Characteristics of vertical stress distribution in surrounding rock outside the lining.

**Fig. 24:**
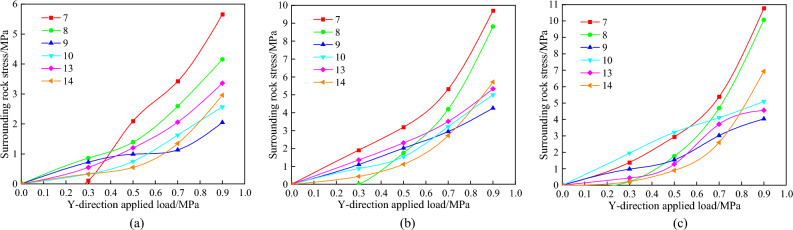
Evolution law of surrounding rock vertical stress under the same lateral pressure coefficient.

The distribution characteristics of surrounding rock vertical stress along the roadway section under equal lateral pressure conditions are shown in Fig. 24, and the stress corresponding to each lateral pressure coefficient increases linearly with the Y-axis load.

The vertical stress in the two sidewall regions increases remarkably. At measuring point 7 (adjacent to the lining), the stress remains stable when the Y-direction load is less than 0.3 MPa, but exhibits an exponential growth trend when the load reaches ≥ 0.5 MPa. For measuring point 8 (located at the far end of the sidewall), the stress accumulates gradually yet remains consistently lower than that at measuring point 7. Notably, the stress difference between these two points converges with the increase in the lateral pressure coefficient, indicating that the stress concentration zone expands outward along the outer side of the lining.

In the roof area, the vertical stress at measuring point 9 shows a continuous upward trend, with significant fluctuations observed when the Y-direction load reaches 0.7 MPa, peaking at 4.04 MPa. In the later stages of loading, the stress at the far-end measuring point 10 surpasses that at measuring point 9.

The stress evolution pattern in the floor area is analogous to that in the sidewall regions. An increase in the lateral pressure coefficient leads to the outward extension of the stress concentration zone, and the stress development exhibits distinct phased characteristics: it increases gently in the early stage, rises sharply within the load range of 0.5 ~ 0.7 MPa, and maintains a high bearing capacity even after lining failure. Overall, the floor stress is lower than that of the sidewalls and approximately equivalent to that of the roof, which verifies the stable controlling effect of the lining on the surrounding rock of the roadway floor.

### Lining internal force distribution characteristics under different lateral pressure coefficients

To explore the influence of lateral pressure coefficient on internal force distribution, based on strain monitoring data, combined with formulas (2) and (3), the bending moment and axial force distribution laws of the prototype structure when the lateral pressure coefficient is 0.8 ~ 1.2 are studied through similarity coefficient conversion. The data are taken from the right half of the structure to investigate the internal force response characteristics under different Y-direction loads.

1. Characteristics of lining bending moment distribution.

**Fig. 25:**
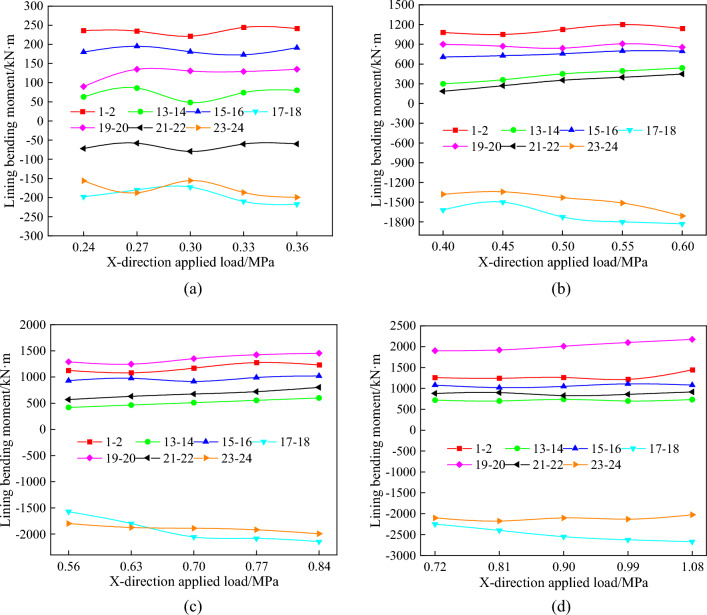
Regulatory effect of lateral pressure ratio on lining bending moment distribution.

As shown in Fig. 25, when the Y-direction load is 0.3 MPa, positive bending moments appear in the 1–2 roof, 13–14 floor middle, 15–16 floor right side, and 19–20 two sidewalls, with the maximum at the arch crown of 244.31 kN m; negative bending moments appear in the 17–18 sidewall foot, 21–22 shoulder, and 23–24 roof right side, with the peak negative bending moment at the arch foot, and the lateral pressure coefficient has little effect on the bending moment, with basically horizontal curves. When the load rises to 0.5 MPa, the bending moment polarity at the arch shoulder reverses, the bending moment increment at the two sidewalls exceeds that at the 15–16 measuring points, and the arch crown increases to 1150.52 kN m. When the load reaches 0.7 MPa and 0.9 MPa, the positive bending moments at the two sidewalls continue to grow and exceed the arch crown, becoming the maximum positive bending moment area.

In summary, when the load and lateral pressure coefficient increase synergistically, the bending moments at all parts of the lining generally increase, and the top and two sidewalls are significantly affected by lateral pressure, which may cause local failure, requiring focused monitoring.

(2) Characteristics of lining axial force distribution.

**Fig. 26:**
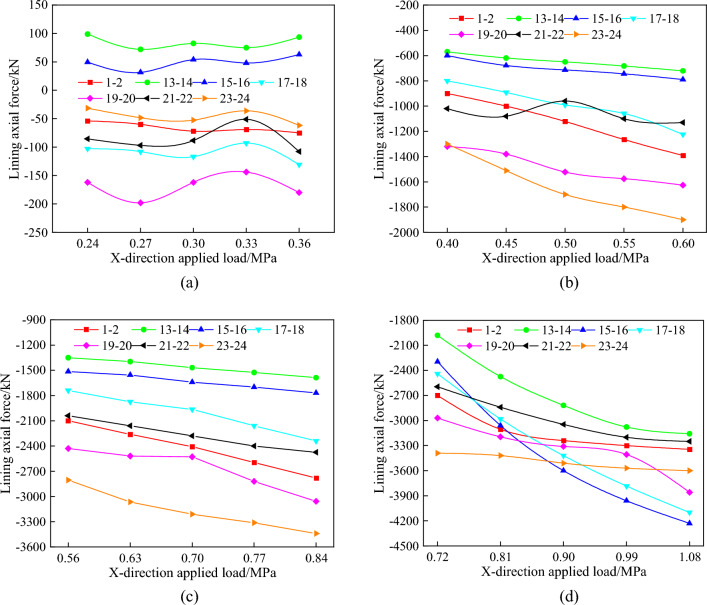
Control effect of lateral pressure ratio on lining axial force distribution.

Figure 26 shows the evolution law of lining axial force under different Y-direction loads. In Fig. 26a, at a Y-direction load of 0.3 MPa, only the floor and right side are under tension, and the rest are under compression; due to insufficient compaction of surrounding rock, the axial force distribution fluctuates significantly, and the fluctuation amplitude is positively correlated with the lateral pressure coefficient. In Fig. 26b and c, at a Y-direction load of 0.7 MPa, the entire section of the structure is under compression, and the increase of lateral pressure coefficient increases the axial force except for the floor and right side; when the lateral pressure coefficient is 1.2, the axial force on the right side of the roof reaches − 3441.086 kN, which is the maximum pressure area. In Fig. 26d, at a Y-direction load of 0.9 MPa, the axial force has a significant linear increase with the lateral pressure coefficient, and the increments at the middle and right side of the floor are the most significant; when the lateral pressure coefficient is 1.2, the axial force at the sidewall foot is − 4140.25 kN, and that at the right side of the floor reaches − 4230.55 kN, which are the pressure extremes simultaneously.

The analysis shows that when the Y-direction load is ≤ 0.5 MPa, the axial force is low and the structure is intact; beyond this threshold, the axial force increments at the right side of the floor and the sidewall foot are significant, forming mechanical weak links, which need to be focused on in engineering.

### Lining internal force distribution laws under the same lateral pressure coefficient

The internal force distribution of the lining structure is not only affected by the lateral pressure coefficient but also by the change of roadway burial depth, which can change its mechanical state. Systematically studying the regulatory law of Y-direction load on lining bending moment and axial force through surface strain monitoring data is the key to analyzing its mechanical response characteristics.

1. Laws of lining bending moment distribution.

**Fig. 27:**
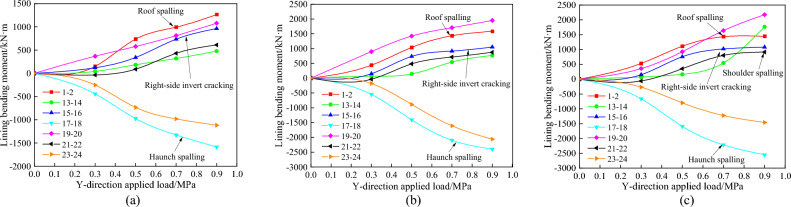
Development law of lining bending moment under constant lateral pressure conditions.

Figure 27 shows the correlation between Y-direction load and lining bending moment. When the lateral pressure coefficient is 0.8, as the Y-direction load increases to 0.3 MPa, the bending moment growth rates of each section differ significantly. Due to the first formation of cracks at the sidewall foot, the bending moment jumps to a peak of − 1584.04 kN m; after the roof cracks, the bending moment growth rate slows down, accumulating to 1265.28 kN m at 0.9 MPa; the crack development and bending moment growth mode on the right side of the floor are similar to those of the arch crown.

When the lateral pressure coefficient is 1.0 ~ 1.2, the bending moment development nodes are advanced and the growth rate is increased, with consistent overall laws; when the coefficient is 1.2 and the load is 0.9 MPa, the shoulder cracking is accompanied by a secondary attenuation of the bending moment growth rate. It can be seen that the increase of Y-direction load drives the linear increase of bending moment, and the increase of lateral pressure coefficient accelerates the bending moment development in key areas.

(2) Characteristics of lining axial force distribution.

**Fig. 28:**
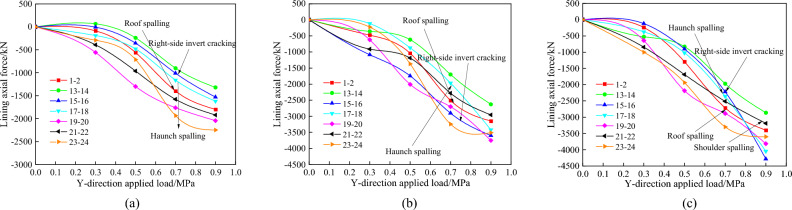
Development law of lining axial force under constant lateral pressure conditions.

Figure 28 illustrates the correlation between the lining axial force and the Y-direction load. When the lateral pressure coefficient is 0.8, as the Y-direction load increases to 0.3 MPa, the axial force in the sidewall foot region surges to − 2044.28 kN due to crack propagation effects. Beyond a Y-direction load of 0.5 MPa, the axial force growth rate on the right side of the floor accelerates, reaching − 1530.55 kN at 0.9 MPa and thus becoming the region with the maximum axial force.

Under a lateral pressure coefficient of 1.0, the lining sustains damage when the Y-direction load reaches 0.5 MPa, leading to a decelerated increase in axial force for both the roof and floor. At the Y-direction load stage of 0.9 MPa, the axial force at the two sidewalls attains − 3751.79 kN, emerging as the area with the highest force.

For a lateral pressure coefficient of 1.2, surface cracks initiate at the arch shoulder under a Y-direction load of 0.9 MPa, with a corresponding axial force of − 3189.86 kN. Meanwhile, the axial force at the two sidewalls continues to rise, reaching − 3815.22 kN at the 0.6 MPa load stage and maintaining the characteristic of being the maximum force-bearing region.

In general, the lining axial force exhibits an overall increasing trend with the enhancement of the Y-direction load. Notably, the axial force at the two sidewalls fluctuates significantly due to early crack initiation. Therefore, in engineering practice, focused attention should be paid to preventing cracking risks induced by excessive axial force in this critical region.

## Conclusions

Through numerical simulations and similar model tests, this paper designs and verifies a high-strength composite support structure suitable for soft rock roadways, and draws the following main conclusions:

1. The numerical simulation results show that there are significant differences in the influences of the burial depth of the high-strength reticulated shell floor, the diameter of the longitudinal reinforcement in the reticulated shell, and the strength of the lining concrete on the stability of deep soft rock roadways. The order of influence is: burial depth of the high-strength reticulated shell floor > strength of the lining concrete > diameter of the longitudinal reinforcement in the reticulated shell. The optimal parameter combination determined through orthogonal tests (with a burial depth of 1.0 m, a longitudinal reinforcement diameter of 12 mm, and a concrete strength of C20) achieves the best control effect, corresponding to a minimum floor heave of 14.32 mm, which can effectively inhibit roadway deformation. Future research could validate this optimal parameter combination in diverse geological settings (e.g., water-rich soft rock or fault zones) to further expand its engineering applicability.
2. The similar model tests reveal the instability characteristics of the support structure. When the Y-direction load increases to 0.9 MPa, the support structure undergoes overall instability due to the propagation of local longitudinal cracks in stress concentration areas such as the sidewall foot and shoulder; however, the “high-strength reticulated shell + shotcrete” composite support structure can delay crack development through synergistic action. It can still maintain good structural integrity when the arch foot stress increases from 4.4  to 9.02 MPa under a 0.7 MPa load, showing significant adaptability to non-uniform stress fields.
3. The stress distribution characteristics show that the surrounding rock of the roof and floor is mainly subject to compressive stress, and there is always stress concentration at the sidewall foot, decreasing from 40.34 MPa to 39.08 MPa; the lining stress is concentrated at the connection between the sidewall foot and the floor, and the peak compressive stress is the lowest at 99.95 MPa when the burial depth is 1.0 m; the steel bars of the high-strength floor reticulated shell mainly bear tensile stress, with the outer secondary arc bars responding significantly, reaching a peak value of 48 MPa, forming a composite bearing system “dominated by compression and coordinated by tension and compression”.
4. Both the model test and numerical simulation results indicate that the high-strength composite support structure can effectively reduce the range of the surrounding rock plastic zone and improve the stress concentration phenomenon by enhancing the floor stiffness through the reticulated shell and enabling the concrete layer to uniformly transmit pressure. It provides a feasible technical solution for the stability control of deep soft rock roadways under complex stress fields, and its parameter optimization method has reference value for similar projects.

## Data Availability

Data will be provided by corresponding author on reasonable request.
